# Multi-zone prediction analysis of city-scale travel order demand

**DOI:** 10.1371/journal.pone.0248064

**Published:** 2021-03-18

**Authors:** Pengshun Li, Jiarui Chang, Yi Zhang, Yi Zhang

**Affiliations:** 1 Tsinghua-Berkeley Shenzhen Institute, Tsinghua University, Shenzhen, PR China; 2 Future Human Habitats Division, Tsinghua Shenzhen International Graduate School, Tsinghua University, Shenzhen, PR China; 3 Department of Mechanical Engineering, Rice University, Houston, Texas, United States of America; 4 Department of Automation, Tsinghua National Laboratory for Information Science and Technology (TNList), Tsinghua University, Beijing, PR China; Monash University, AUSTRALIA

## Abstract

Taxi order demand prediction is of tremendous importance for continuous upgrading of an intelligent transportation system to realise city-scale and personalised services. An accurate short-term taxi demand prediction model in both spatial and temporal relations can assist a city pre-allocate its resources and facilitate city-scale taxi operation management in a megacity. To address problems similar to the above, in this study, we proposed a multi-zone order demand prediction model to predict short-term taxi order demand in different zones at city-scale. A two-step methodology was developed, including order zone division and multi-zone order prediction. For the zone division step, the K-means++ spatial clustering algorithm was used, and its parameter k was estimated by the between–within proportion index. For the prediction step, six methods (backpropagation neural network, support vector regression, random forest, average fusion-based method, weighted fusion-based method, and k-nearest neighbour fusion-based method) were used for comparison. To demonstrate the performance, three multi-zone weighted accuracy indictors were proposed to evaluate the order prediction ability at city-scale. These models were implemented and validated on real-world taxi order demand data from a three-month consecutive collection in Shenzhen, China. Experiment on the city-scale taxi demand data demonstrated the superior prediction performance of the multi-zone order demand prediction model with the k-nearest neighbour fusion-based method based on the proposed accuracy indicator.

## Introduction

Traffic has become an important factor impacting city management and operation as well as the daily lives of numerous dwellers. One of the most fundamental problems for a smart city is identifying a method to establish an efficient transportation system. Currently, with the continuous upgrading of intelligent transportation systems to realise city-scale services and personalised services, taxi demand data are of concern and need to be accessed increasingly rapidly. Moreover, directly processing city-scale data could pressurise data-processing systems, because the data may be highly complex, posing tremendous challenges for the real-time calculation of city-scale system operation management. To address this problem, a critical approach is accurate short-term multi-zone taxi demand prediction by dividing the order zone to reduce the processing time and improve the prediction accuracy simultaneously. An accurate prediction of the taxi order demand implies the provision of assistance to a city to pre-allocate resources and facilitation of city-scale taxi operation management in a megacity [1]. This type of a model can be beneficial to numerous city-scale operational management scenarios. For example, for a sharing service, it can help facilitate the schedule of sharing the vehicle fleet in advance to reduce the high cruise expense. Further, for taxi operation management, it can reduce the imbalance between the taxi supply and demand in some areas.

The literature includes numerous studies of prediction methods under different traffic scenarios, including traffic volume, taxi demand, and traffic flow volume. Time series analysis methods are the most prominent models for the prediction of traffic. Representatively, the autoregressive integrated moving average (ARIMA) is a well-known time series forecasting model, owing its short-term prediction performance [2–4]. For example, Li et al. [4] used an ARIMA variant to predict the number of passenger pick-ups from urban hotspots. More recently, machine learning methods are being frequently used to predict future traffic data, which attempt to identify historical data that are similar to the prediction instant. These methods include neural networks (NNs), support vector regression (SVR), random forest (RF), and k-nearest neighbour (kNN). Mukai et al. [5] predicted taxi demand from the taxi probe data of Tokyo using a back-propagation neural network. Feng et al. [6] proposed a novel improved support vector machine (SVM) prediction algorithm to predict short-term traffic flow subsequent to the use of the adaptive particle swarm optimization algorithm, to optimize the parameters of the above-mentioned improved SVM. Nikravesh et al. [7] compared some machine learning methods based on the network traffic data, and the results demonstrated that the SVM performed better in predicting the multidimensionality of network traffic data. Habtemichael et al. [8] proposed an enhanced kNN algorithm for short-term traffic forecasting, and it was found to provide promising results. More recently, deep learning methods, such as convolutional neural networks (CNNs) and long short-term memory networks (LSTMs) have been applied to short-term traffic prediction [9, 10]. Ma et al. [9] employed a CNN to predict large-scale network-wide traffic speed by learning traffic as images, and the result demonstrated that this method could outperform other existing algorithms by a large accuracy improvement. Yu et al. [10] built a deep LSTM to predict the peak-hour traffic of a large-scale traffic flow in Los Angeles, following identification of the unique characteristics of the traffic data.

Although individual prediction methods present good predictive performances, to further reduce the prediction error, in recent years, some researchers have attempted to combine prediction models to improve the prediction accuracy [11, 12]. Qiu et al. [13] proposed an integrated precipitation-correction model to use the fusion method with four prediction models to predict freeway traffic flow. Vlahogianni [1] combined three different prediction models to propose a surrogate model for freeway traffic speed prediction. Moreover, these studies verified that a fusion-based prediction model could improve the prediction accuracy.

From the literature, it can be found that numerous studies on taxi order demand prediction methods are predominantly concerned about temporal changes. To increase the prediction performance and to adapt to city-scale development, some studies consider the spatial effect on the former. For example, some researchers investigate taxi order demand prediction for hotspot analysis or grids. Li et al. [4] analysed the spatial–temporal variation of passengers in a hotspot. Chang et al. [14] used a clustering method to identify a hotspot area and then predicted its traffic data. Ke et al. [15] employed a novel deep learning approach called the fusion convolutional LSTM network to predict the passenger demand for on-demand ride services for 7×7 grids in Hangzhou, China by analysing spatial–temporal characteristics. Yao et al. [16] utilized a CNN and a LSTM to predict the taxi order demand for 20×20 grids. Currently, although most studies focus on hotspots or uniformly divided grid zones, there are no investigations on multi-zone prediction analysis considering the order spatial distribution as well as on the overall performance for zone prediction.

In this study, we develop a multi-zone order demand prediction model to conduct a spatial–temporal prediction analysis. Specifically, it aims at predicting the order demand in different parts of an urban area by considering the traffic prediction and spatial difference simultaneously. The model reuses order information of the current time period continuously sent/received by the telematics systems installed on all the taxis to predict the order demand of the next period in different zones, which are divided by a clustering algorithm based on data similarity. The main contributions of this study are as follows:

1. The proposed two-step model composed of a clustering-based multi-zone division model and six prediction methods (backpropagation neural network (BP–NN), SVR, RF, average fusion-based method, weighted fusion-based method, and kNN fusion-based method) can realise spatial–temporal prediction at city-scale. The clustering-based multi-zone division model uses the K-means++ clustering algorithm, and the above six prediction methods are employed for the order prediction and comparison analysis.
2. The proposed multi-zone order demand prediction model is validated by real-world taxi order demand data in Shenzhen, China.
3. A systematic comparison analysis is developed to compare the prediction accuracies of the six prediction methods and the prediction accuracies of the two different zone division methods, the proposed clustering based zone division method and the commonly used grid zone division method. Furthermore, we propose multi-zone weighted indicators to evaluate the overall prediction performances of these prediction models.

The remainder of the paper is organized as follows. Section 2 describes the structure and mathematical formulation of the proposed multi-zone taxi order demand prediction model. Section 3 presents the analyses of the prediction performance of the multi-zone taxi order demand prediction model based on a real-world dataset. Finally, we conclude the paper in Section 4.

## Methodology

**Fig 1:**
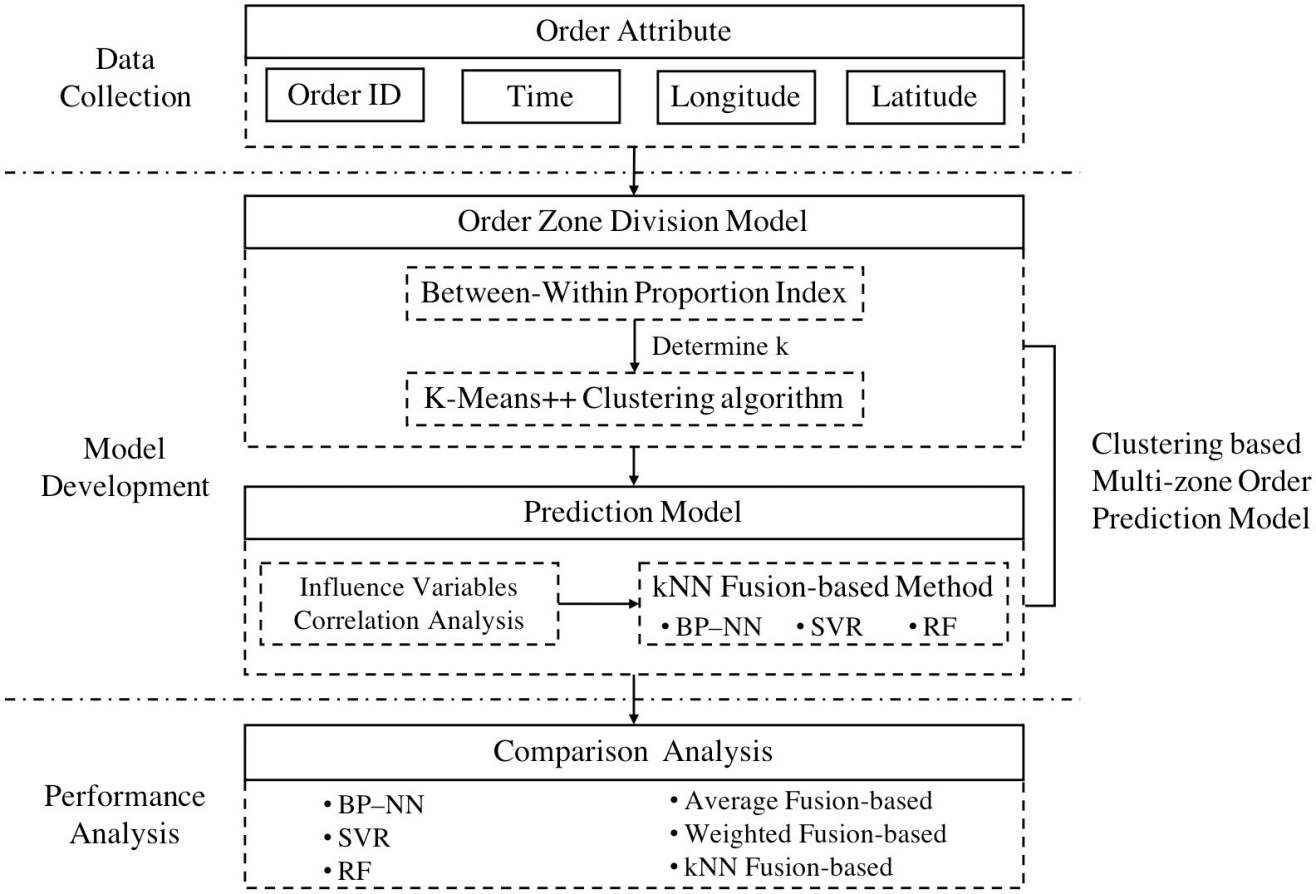
Research framework.

The proposed clustering-based multi-zone order prediction model aims to predict short-term taxi order demand in multiple zones. The framework of this model is shown in Fig 1. For data collection, we collect information including the order ID, order time, order longitude, and order latitude, and these could provide an adequate basis for the model and analysis discussed below. For the model development, we propose a clustering based multi-zone order prediction model to predict short-term order demand in multiple zones. This model contains two parts. The first model is the order zone division model. In this model, we first propose a cluster validity index called the between–within proportion (BWP) index to determine the optimal number (k) of zones, an important parameter of the K-means++ clustering algorithm. Then we use the K-means++ clustering algorithm to divide the entire order zone. The second model is the prediction model. In this model, first we conduct influence variable correlation analysis to determine the input variables of the prediction model, and then we use cross validation to estimate the parameter for the prediction model. Subsequently, we propose six prediction models: BP–NN, SVR, RF, average fusion-based method, weighted fusion-based method, and kNN fusion-based method for taxi order prediction. The three fusion-based methods combine the predicted outputs of the BP-NN neural network, SVR, and RF based on some principles to yield the final prediction results. For the performance analysis, we compare the prediction performance of these six different prediction models in different zones by using prediction accuracy indicators. These include the mean absolute error (MAE), mean absolute percentage error (MAPE), root mean square error (RMSE). Further, three multi-zone weighted accuracy indicators, including the multi-zone weighted MAE (MZW-MAE), multi-zone weighted MAPE (MZW-MAPE), and multi-zone weighted RMSE (MZW-RMSE) are employed to compare the overall performances of these prediction models.

### Order zone division model

Order demand varies with the area. To better analyse and predict taxi demand, it is necessary to divide an area into several zones. However, dividing the area based on administrative districts or uniformly grid division cannot accurately reflect the spatial distribution difference in taxi demand. Further, spatial clustering algorithms, whose clustering principle is based on data similarity could better reflect this difference.

### Spatial clustering algorithm

Currently, the well-known spatial clustering algorithms are K-means, density-based spatial clustering of applications with noise (DBSCAN), and other spatial algorithms. The K-means clustering algorithm aims to partition the n observations into k clusters in so as to minimize the within-cluster sum of squares [17]. The DBSCAN is a typical density clustering algorithm which clusters based on the region density, and the region density must exceed the predefined density threshold in the given radius neighbourhood, so that it can find clusters of any shape [18]. However, the DBSCAN algorithm is slightly more complex than the K-means algorithm. It needs to coordinate the neighbourhood sample number threshold and the distance threshold, ϵ. The final clustering effect differs based on the combination of different parameters. In addition, the dataset can typically be extremely large, so that when the DBSCAN clustering algorithm is used, its convergence time can be significantly long. Conversely, the K-means algorithm is more suitable for large datasets, and the algorithm has relative scalability and high efficiency. Its time complexity is *0*(*nkt*), where n is the number of samples, k is the number of clusters, and t is the number of iterations.

The choice of the k initial centroids directly affects the accuracy of the final clustering result and the duration of the algorithm operation; therefore, it is necessary to select the appropriate k centroids. K-means++ algorithm optimizes the initialization centroids based on the K-means algorithm. Therefore, it is chosen to divide the order zone.

The principle of the K-means algorithm is that given a set of data points, $\chi =\{x_{1},\ldots ,x_{n}\{,x_{n}\in R^{n}$, the K-means clustering algorithm partitions the input into *k* ≤ *m* sets, *C*_1_,…,*C*_*k*_ to minimize the within-cluster sum of squares (WCSS) given by:

$$\underset{C}{\text{argmin}}\sum_{j=1}^{k}\sum_{x\in C_{j}}∥x-\mu_{j}{∥}^{2},$$ (1)

where *μ*_*j*_ denotes the jth centroid, *c*^(*i*)^ ∈ {1,…,*k*} denotes the cluster label for *x*_*i*_, and ‖⋅‖^2^ is the square of the Euclidean distance.

The K-means algorithm flow is as follows:

**Step1**: Initialize cluster centroids $\mu_{1},\ldots ,\mu_{k}\in R^{n}$ randomly from *χ*.
**Step2**: For every *i*, assign *x*_*i*_ to the cluster with the closest centroid, in other words, *c*^(*i*)^ = argmin_*j*_ ‖*x*_*i*_ − *μ*_*j*_‖^2^.
**Step3**: For every *j*, update centroid *μ*_*j*_, set *μ*_*j*_ to be the center of mass of all points in $C_{j}:\mu_{j}=\frac{1}{\left|C_{j}\right|}\sum_{x\in C_{j}}x$.
**Step4**: calculate the deviation, $D=\sum_{j=1}^{k}\sum_{x\in C_{j}}{\left\|x-\mu_{j}\right\|}^{2}$.
**Step5**: repeat Step 2, 3 and 4 until *D* converges.

The K-means++ clustering algorithm uses a distance-based sampling method to optimize the initialization centroids based on the K-means algorithm. The K-means++ algorithm flow is as follows:

**Step1a**: Take one centroid *μ*_1_, chosen uniformly at random from *χ*.
**Step1b**: Take a new centroid *μ*_*j*_, choosing *x* ∈ *χ* with probability $\frac{{D(x)}^{2}}{\sum_{x\in \chi }{D(x)}^{2}}$.
**Step1c**: Repeat Step 1b until we have taken *k* centroids altogether.
**Step2-5**: Proceed as with the standard K-Means algorithm.

#### Determination of parameter k

The most critical part of the K-means++ algorithm is the determination of the value of k. However, in reality, k is difficult to be accurately determined. The indexes used to test the validity of the clusters have been proposed by scholars from various countries, including the Calinski–Harabasz index (CH), Davies–Boudin index (DB), Krzanowski–Lai index (KL), weighted inter-intra index (Wint), in-group proportion index (IGP), and others [19, 20]. Using these indexes to calculate the clustering validity of k clusters in the range of [*k*_*min*_, *k*_*max*_], the optimal number of clusters, *k*_*opt*_, can be obtained. However, these indexes have defects. When the clustering structure cannot be discriminated, the test results are not sufficiently ideal; therefore, it is difficult to obtain a perfect optimal number of clusters. Zhou et al. [21] proposed a new index according to the geometric structure of the sample, and after a large amount of data verification, the clustering result obtained by this index improved. Thus, for the determination of the value of k, this study chooses the BWP index.

Let *k* = {*X*, *R*} be the clustering space, where *X* = {*x*_1_, *x*_2_,…,*x*_*n*_}, n is the number of samples, and c is the number of clusters.

*Definition 1*. Let the minimum interclass distance, *b*(*i*, *j*), of the *j*th sample of the *i*th class be expressed as the minimum of the average distance of the sample to all the samples in each of the other classes, which is given by:

$$b(i,j)=\underset{1\leq k\leq c,k\neq i}{\text{min}}(\frac{1}{n_{k}}\sum_{p=1}^{n_{k}}∥x_{p}^{\left(k\right)}-x_{j}^{\left(i\right)}{∥}^{2}),$$ (2)

where k and i are class labels, $x_{j}^{\left(i\right)}$ is the *j*th sample of the *i*th class, $x_{p}^{\left(k\right)}$ is the *p*th sample of the *k*th class, *n*_*k*_ is the number of samples in the kth class, and ‖⋅‖^2^ is the square Euclidean distance.

*Definition 2*. Let the minimum intra-class distance, *w*(*i*, *j*), of the *j*th sample of the *i*th class be expressed as the average distance of the sample to all the other samples in the *i*th class, which is given by:

$$w(i,j)=\frac{1}{n_{i}-1}\sum_{q=1,q\neq j}^{n_{i}}∥x_{q}^{\left(i\right)}-x_{j}^{\left(i\right)}{∥}^{2},$$ (3)

where $x_{q}^{\left(i\right)}$ is the *q*th sample of the *i*th class, and *q* ≠ *i*, *n*_*i*_ is the number of samples in the *i*th class.

*Definition 3*. Let clustering distance, *baw*(*i*, *j*), of the *j*th sample of the *i*th class be expressed as the sum of the minimum inter-class distance and intra-class distance of the sample, which is given by:

bsw(i,j)=b(i,j)+w(i,j)

$$=\underset{1\leq k\leq c,k\neq i}{\text{min}}(\frac{1}{n_{k}}\sum_{p=1}^{n_{k}}∥x_{p}^{\left(k\right)}-x_{j}^{\left(i\right)}{∥}^{2})+\frac{1}{n_{i}-1}\sum_{q=1,q\neq j}^{n_{i}}∥x_{q}^{\left(i\right)}-x_{j}^{\left(i\right)}{∥}^{2},$$ (4)

*Definition 4*. Let clustering subtraction distance, *bsw*(*i*, *j*), of the *j*th sample of the *i*th class be expressed as the difference between the minimum inter-class distance and intra-class distance of the sample, given by:

bsw(i,j)=b(i,j)+w(i,j)

$$=\underset{1\leq \text{k}\leq \text{c},\text{k}\neq \text{i}}{\text{min}}(\frac{1}{{\text{n}}_{\text{k}}}\sum_{\text{p}=1}^{{\text{n}}_{\text{k}}}∥{\text{x}}_{\text{p}}^{\left(\text{k}\right)}-{\text{x}}_{\text{j}}^{\left(\text{i}\right)}{∥}^{2})+\frac{1}{{\text{n}}_{\text{i}}-1}\sum_{\text{q}=1,\text{q}\neq \text{j}}^{{\text{n}}_{\text{i}}}∥{\text{x}}_{\text{q}}^{\left(\text{i}\right)}-{\text{x}}_{\text{j}}^{\left(\text{i}\right)}{∥}^{2},$$ (5)

*Definition 5*. Let the Between-Within Proportion index, *BWP*(*i*, *j*), of the *j*th sample of the *i*th class be expressed as the ratio of the clustering subtraction distance to the clustering distance of the sample (see Eq (6)):

$$BWP(i,j)=\frac{bsw(i,j)}{baw(i,j)}=\frac{b(i,j)-w(i,j)}{b(i,j)+w(i,j)}$$

$$=\frac{\underset{1\leq k\leq c,k\neq i}{\text{min}}(\frac{1}{n_{k}}\sum_{p=1}^{n_{k}}∥x_{p}^{\left(k\right)}-x_{j}^{\left(i\right)}{∥}^{2})-\frac{1}{n_{i}-1}\sum_{q=1,q\neq j}^{n_{i}}{∥x_{q}^{\left(i\right)}-x_{j}^{\left(i\right)}∥}^{2}}{\underset{1\leq k\leq c,k\neq i}{\text{min}}(\frac{1}{n_{k}}\sum_{p=1}^{n_{k}}∥x_{p}^{\left(k\right)}-x_{j}^{\left(i\right)}{∥}^{2})+\frac{1}{n_{i}-1}\sum_{q=1,q\neq j}^{n_{i}}{∥x_{q}^{\left(i\right)}-x_{j}^{\left(i\right)}∥}^{2}},$$ (6)

From the perspective of the intra-class distance, the smaller the value of *w*(*i*, *j*), the better the result. From the perspective of the inter-class, the larger the value of *b*(*i*, *j*), the better the result. To achieve equilibrium in both the cases, a linear combination is chosen to consider both requirements. Clustering subtraction distance *bsw*(*i*, *j*) = *b*(*i*, *j*) + (−*w*(*i*, *j*)) can be used to evaluate the clustering result. The larger the value of *bsw*(*i*, *j*), the better the clustering effect. Simultaneously, to reduce the influence of dimension on clustering, clustering distance *baw*(*i*, *j*) is introduced. The index can be made dimensionless by compressing *bsw*(*i*, *j*) by *baw*(*i*, *j*); thus, the range of the values of the index is [-1,1]. If *BWP*(*i*, *j*) ≈ 1, it indicates that the sample is correctly clustered. If *BWP*(*i*, *j*) ≈ −1, it indicates that the sample is incorrectly clustered.

Index *BWP*(*i*, *j*) only reflects the clustering of a certain sample; it does not reflect the clustering of all the samples. However, if the average of the *BWP*(*i*, *j*) of all the samples in the dataset is obtained, the clustering effect of the dataset can be reflected. The larger the average value, the better the clustering effect of the dataset, and the number of clusters corresponding to the maximum value is the optimal clustering number, which is given by

$$avg_{BWP}(k)=\frac{1}{n}\sum_{j=1}^{k}\sum_{i=1}^{n_{j}}BWP(i,j),$$ (7)

$$k_{opt}=\underset{2\leq k\leq n}{\text{argmax}}\{avg_{BWP}(k)\},$$ (8)

where *avg*_*BWP*_(*k*) is the average value of the BWP obtained when the sample set is clustered into k classes and *k*_*opt*_ is the optimal clustering number.

### Order prediction model

The BP–NN, SVR, and RF are adopted in this study, because they are commonly used prediction models. Furthermore, the average fusion-based method, weighted fusion-based method, and kNN fusion-based method are also employed in this study for their advantages of effectively reduction in the large error of an individual prediction model in a certain prediction [22].

#### Backpropagation neural network

A BP neural network is a multi-layer feedforward neural network based on the error back propagation [23]. The basic concept is to use the network MSE as the objective function. Based on the gradient descent strategy, the parameters are adjusted in the negative gradient direction of the target to minimize the MSE between the expected output value and the true value. The BP neural network model has the advantage of approximating non-linear functions with arbitrary precision. In this study, cross-validation is performed for the selection of parameters. After minimizing the MAPE in the process of optimization, the number of hidden layer can be determined.

#### Support vector regression

SVR is a well-known machine learning method that emerged at the end of the 20th century. It is mainly used for classification and prediction and has good generalization ability. It was proposed by the world-renowned scholar Vapnik [24]. SVR continuously adjusts the parameters by training the samples to derive a model that minimizes the sum of the deviations between the predicted and the true values of all the training samples. By inputting the predicted input vector into the model, the value can be predicted. In this study, a radial basis function (RBF) is utilized as the kernel function because it is demonstrated to be highly suitable for traffic prediction under different conditions [12]. After optimising, the capacity values, C, of the SVR can be determined, and in this study, the *ε*-insensitive loss function is used.

#### Random forest

Decision trees are a well-known classification and regression method owing to their simplicity and ease of implementation. However, they do not usually have a good predictive performance as a result of being prone to overfitting. To enhance this traditional method, random forest (RF) has been used as an ensemble prediction model that produces a large number of decision trees in parallel to reduce the bias and variance of the predictions [25]. In the tree construction process of an RF, there are two primary statistical techniques involved: bootstrapping and bagging. First, through randomly sampling the entire training data set with replacement, RF generates N bootstrapped training data sets. Each bootstrapped training data set is employed to construct a regression tree. During the training process, it is possible that the constructed decision trees are highly correlated, especially when the root node is a relatively strong variable. For this reason, rather than consider all the variables, only a small, fixed number, m, of the total variables are considered at each split. After optimisation, m can be determined. Subsequently, during the prediction, all N trees are bagged to have an average value that reduces the variance of the model.

#### Average fusion-based method

Average fusion-based method: The average of the prediction results of each individual predictor is taken as the final result. Given a set of predictors, ${\hat{y}}^{i},i=1,\ldots ,m$, we seek to compute final prediction $\hat{y}$ as follows:

$$\hat{y}=\frac{1}{m}({\hat{y}}^{1}+{\hat{y}}^{2}+\cdots +{\hat{y}}^{m}),$$ (9)

where ${\hat{y}}^{i}$ is the prediction result using the *i*^*th*^ predictor, *m* is the total number of the predictors. In this study, the predictors are BP–NN, SVR, and RF, thus, m is set to 3.

#### Weighted fusion-based method

In this method, the weights of different predictors are not identical. The weighted hybrid method is written as:

$$\hat{y}=\alpha_{1}{\hat{y}}^{1}+\alpha_{2}{\hat{y}}^{2}+\cdots +\alpha_{m}{\hat{y}}^{m}=\sum_{i}\alpha_{i}{\hat{y}}^{i},$$ (10)

$$\alpha_{i}=\frac{1/MAPE_{i}}{\sum_{j}1/MAPE_{j}},$$ (11)

where ${\hat{y}}^{i}$ is the prediction result using the *i*^*th*^ predictor and *α*_*i*_ is the weight of the *i*^*th*^ predictor. In this study, the weights are generally calculated by the inverse of the MAPE using the training dataset.

#### kNN fusion-based prediction model

**Fig 2:**
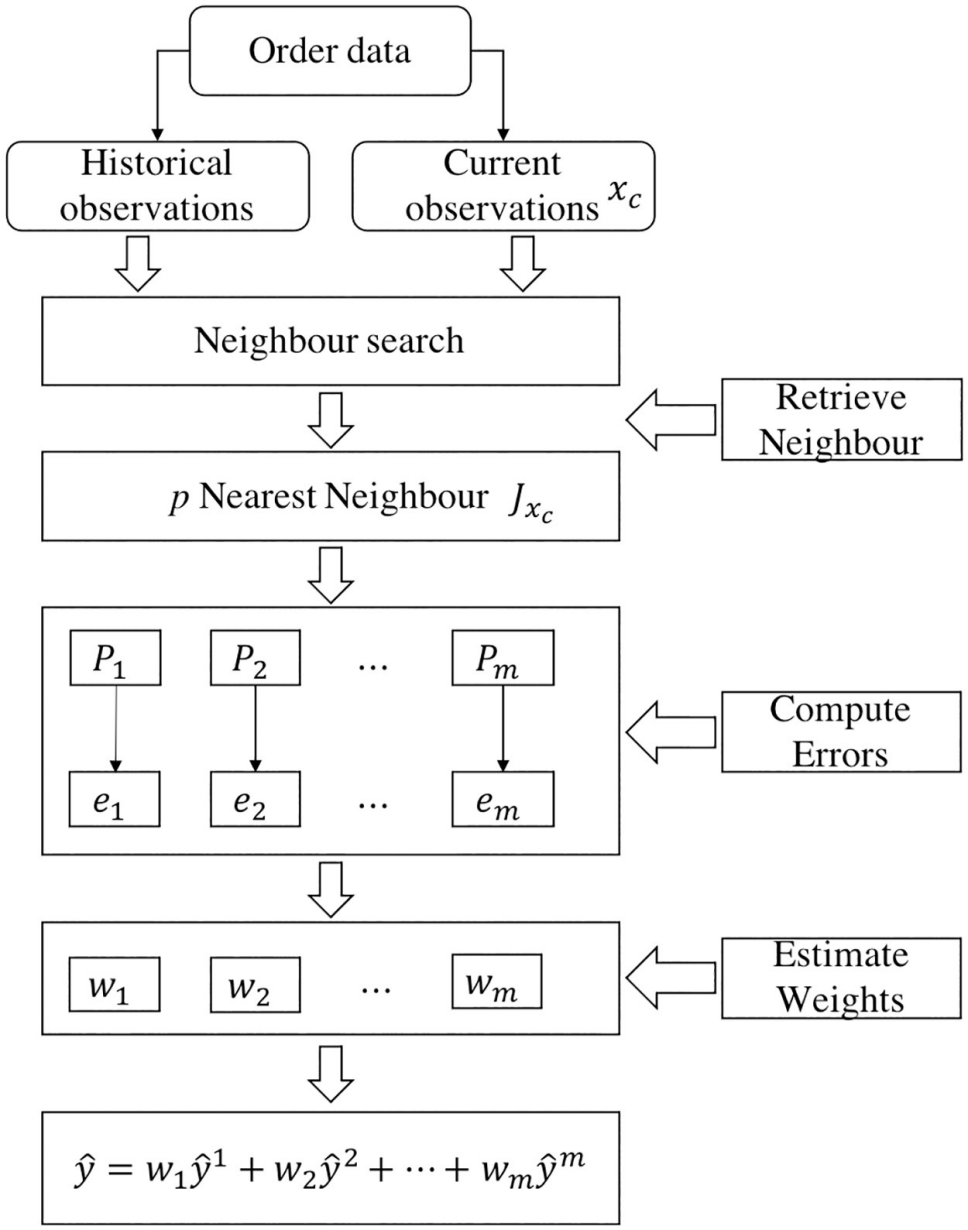
Flowchart based on the kNN fusion-based method.

The kNN fusion-based method is highly unstructured and does not require any pre-determined model specification. The basic concept of the kNN fusion-based method is that in the scenario of the current traffic state, search the nearest neighbour to this state in the training used historical datasets, compute the prediction errors of the nearest neighbour set, estimate the weights of each predictor, and combine the final predicted outputs of each individual predictor based on these weights [26]. Fig 2 depicts the flowchart of the kNN fusion-based method. There are two steps in the kNN fusion-based method.

*Step 1*: *Neighbourhood searching process*. The search process finds the nearest neighbours, which are the historical observations that are most similar to the current observation. The Euclidean distance is used in this study to determine the distance between the current input feature vector and the historical observations. *p* is the number of historical observations with the nearest distances to the input feature vector. The set of *p* nearest neighbours of the input feature vector *x*_*c*_ can be written as $J_{x_{c}}={\left[x_{1},x_{2},\ldots ,x_{p}\right]}^{T}$ and ***x***_*j*_ = [*x*_*j*1_, *x*_*j*2_,…*x*_*jn*_], where *j* = 1,2,…,*p* and *n* is the dimension of the feature space. The trial and error method introduced by Guo et al. [27] is used for setting the parameters of the kNN. In this study, *p* is set to 5.

*Step 2*: *Weighted parameter estimation process*. This process is used to calculate the weights of each predictor. For each vector ***x***_*j*_, the predicted value, ${\hat{y}}_{j}^{i}=f_{i}(x_{j})$, where *i* = 1,2,…,*m*, *f*_*i*_ denotes the *i*^th^ predictor and error $e_{j}^{i}={\hat{y}}_{j}^{i}-y_{j}$, of each predictor can be calculated. Hence, the errors can be used to estimate the weights of each predictor at the current time as *w*_*i*_ = (1/MAPE_*i*_)/∑_*j*_(1/MAPE_*j*_), where the MAPE is calculated based on the selected nearest neighbour dataset. The main difference between the weighted fusion-based method and the kNN fusion-based method is that the weights used in the latter are dynamically updated in each step.

### Prediction accuracy indicators

To better compare and analyse the actual prediction performance and the effects of different prediction models, the predictors need to be evaluated and analysed. In this study, the following performance evaluation indicators are adopted: MAE, MAPE, and RMSE, which are given by:

$$MAE=\frac{1}{N}\sum_{i}|{\hat{D}}_{i}-D_{i}|,$$ (12)

$$MAPE=\frac{1}{N}\sum_{i}\frac{\left|{\hat{D}}_{i}-D_{i}\right|}{D_{i}},$$ (13)

$$RMSE=\sqrt{\frac{\sum_{i}{\left[{\hat{D}}_{i}-D_{i}\right]}^{2}}{N}},$$ (14)

where *N* is the total sample size, ${\hat{D}}_{i}$ is the predicted order value, and *D*_*i*_ is the observed value. Since MAPE is greatly affected by the very small values, we only calculate MAPE for samples with taxi demand higher or equal to 5. This is a common practice used in many studies, as low-demand scenarios can be less concerned [16, 28].

Based on the above indicators, the predicted results of the BP–NN, SVR, RF, average fusion-based, weighted fusion-based, and kNN fusion-based prediction models are analysed and evaluated.

To better compare the performances of the different prediction models in terms of multi-zone prediction, we then proposed multi-zone weighted indicators based on the MAE, MAPE, and RMSE to evaluate the overall prediction performances of these prediction models in all the zones. These included MZW-MAE, MZW-MAE, and MZW-RMSE, which are given by:

$$MZW-MAE=\sum_{k}\frac{o_{k}}{O}MAE_{k},$$ (15)

$$MZW-MAPE=\sum_{k}\frac{o_{k}}{O}MAPE_{k},$$ (16)

$$MZW-RMSE=\sum_{k}\frac{o_{k}}{O}RMSE_{k},$$ (17)

where *O* is the total number of order demands in all the zones in the testing dataset, *o*_*k*_ is the number of order demands in zone *k*, and *MAE*_*k*_, *MAPE*_*k*_, *RMSE*_*k*_ are the prediction accuracy indicators for zone *k*.

## Numerical experiment

### Data description and processing

The data in this study are provided by DiDi-UDian Scientific Shenzhen Co., which are collected from 10 August to 23 October 2015 in Shenzhen, China (https://github.com/thu-lps/taxi-demand-order.git). The order data include information such as the order ID, order time, order longitude, and latitude. (Ethic committee of Tsinghua University Shenzhen International Graduate School confirmed that the ethic approval was waived in this case.).

**Fig 3:**
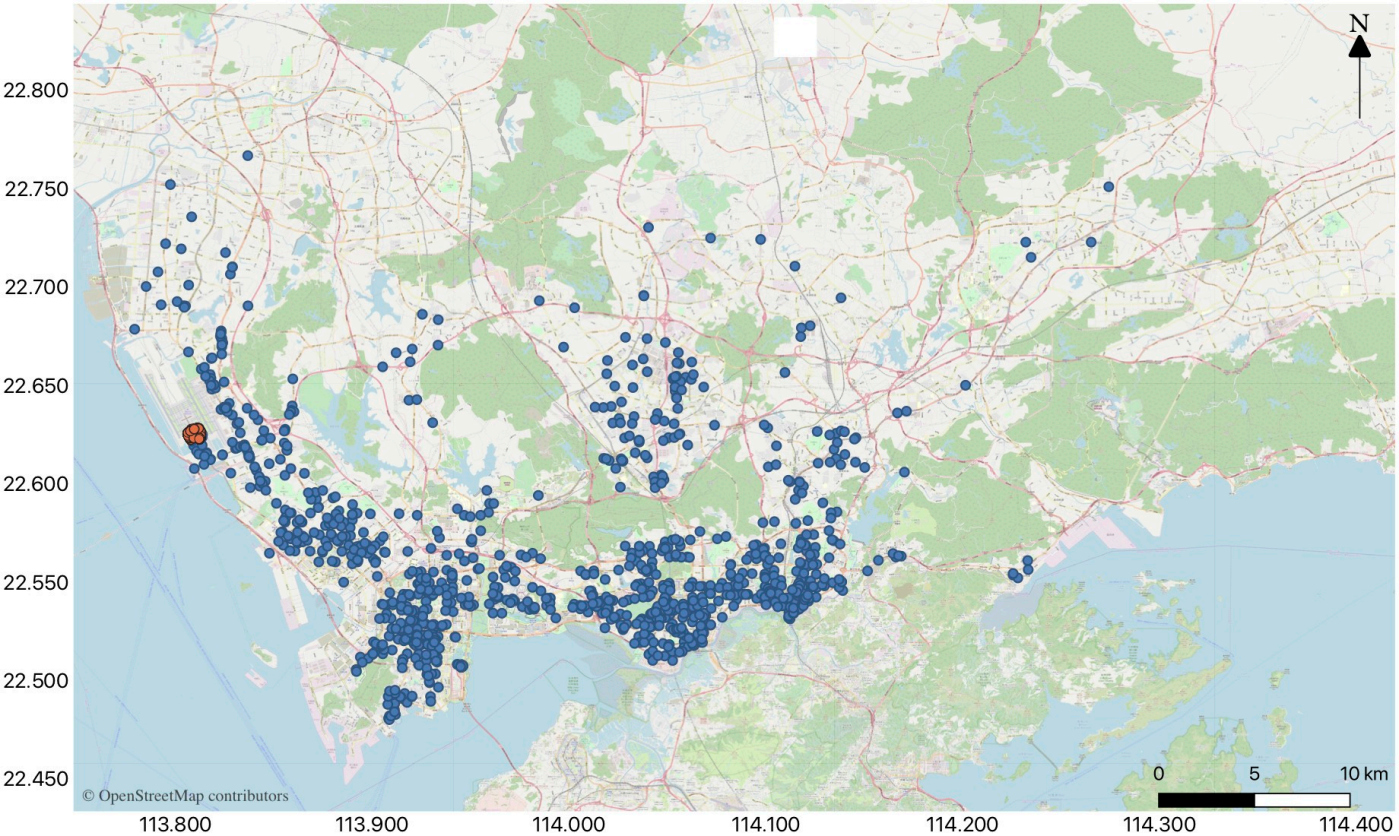
Visualization of the order demand on 10 August, 2015 (orders to Shenzhen Airport).

The aim of this study is to adopt the order prediction in the Shenzhen urban area to Shenzhen Airport, for example, and it does not consider the order demand outside the city. The coordinate range of Shenzhen is 22°45′~22°82′ north latitude, and 113°71′~114°37′ east longitude, which is the area of the study zone. Then the data in the above range are selected. Furthermore, as we predict the taxi order demand to the airport, it is necessary to extract the order data for which the order destination points are Shenzhen Airport. The extraction results are visualized as shown in Fig 3. (blue points are the order origin points and the orange points are the order destination points at Shenzhen Airport).

### Clustering-based zone division

The order demands to Shenzhen Airport differ between working days and non-working days, and passenger taxi demands have a strong regularity over working days. Therefore, this section only divides the order zone of the working days as the case study. As the daily taxi order to the airport is relatively low, in this study, the GPS data of all the Shenzhen taxi orders in the working days spanning from 1 September 2015 to 31 September 2015, including the longitude and latitude of the data, were used for zone division to reduce the randomness caused by the small amount of data and data dispersion.

**Fig 4:**
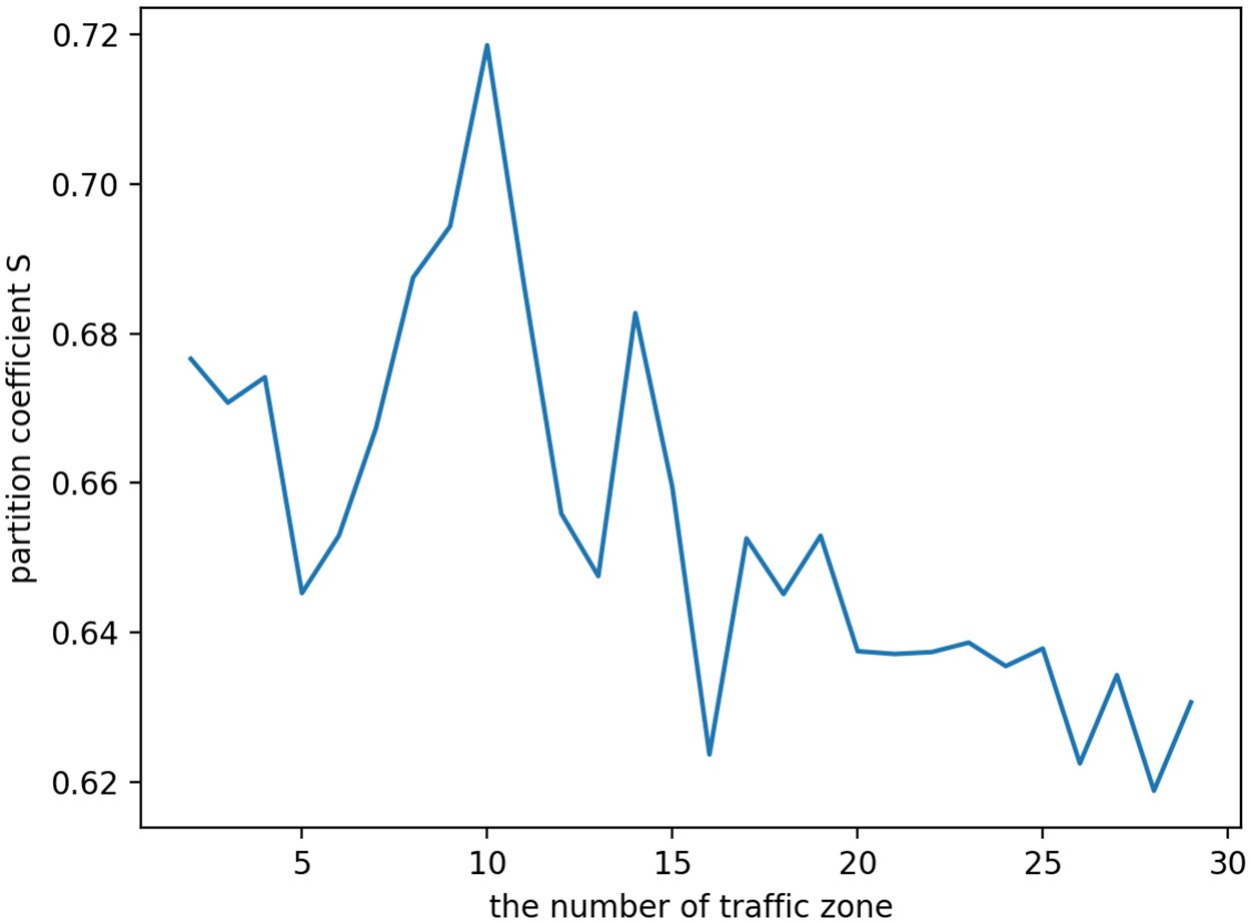
Curve of the BWP value variation with the k value.

The range of the values of k is limited to [2,30] based on experience and the actual conditions. After calling the K-means++ algorithm, the k-value curve is obtained (shown in Fig 4).

As can be seen from Fig 4, when the value of k is 10, the value of the BWP index is the largest, which is 0.71826. Hence, the number of order areas in this study is determined to be 10.

**Fig 5:**
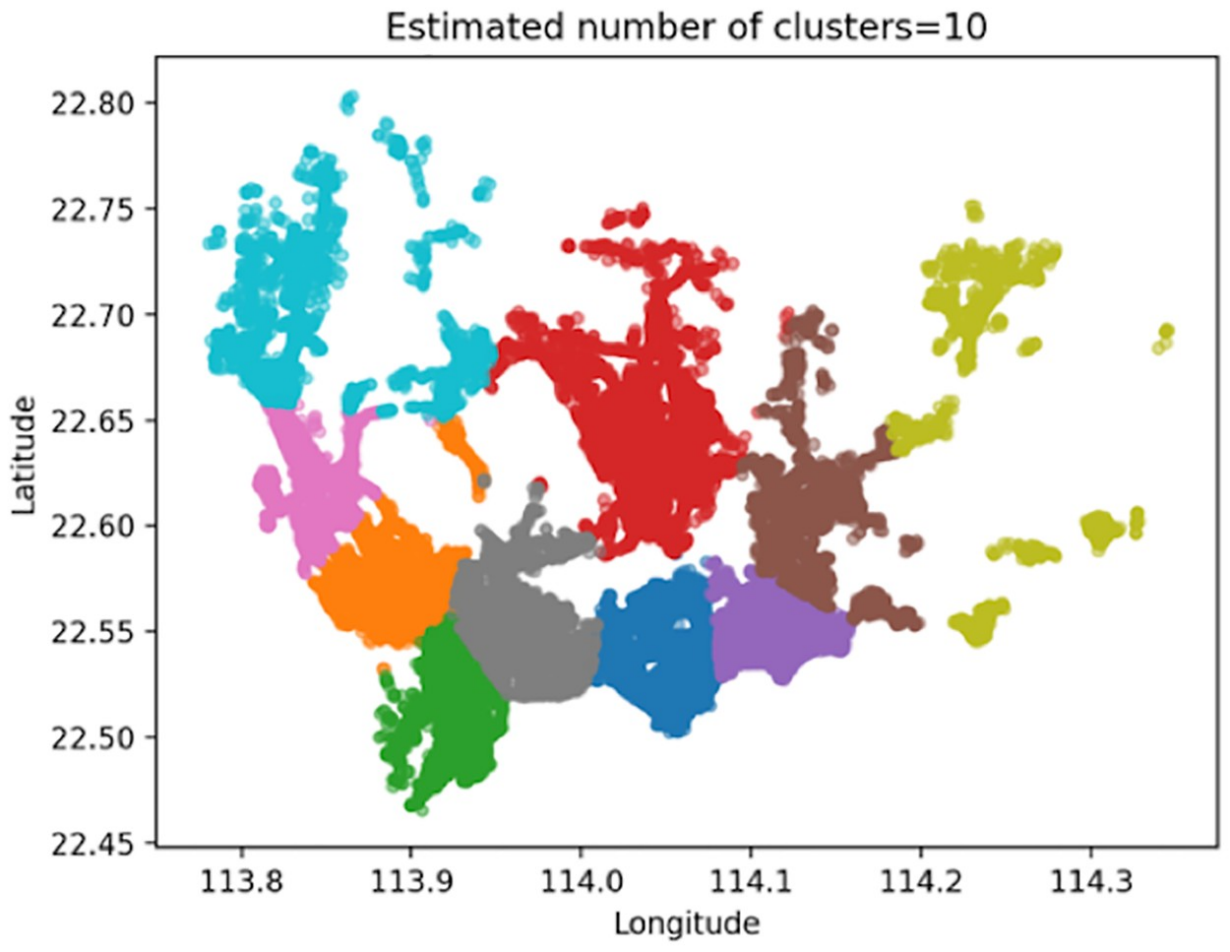
Clustering result graph with k = 10.

**Fig 6:**
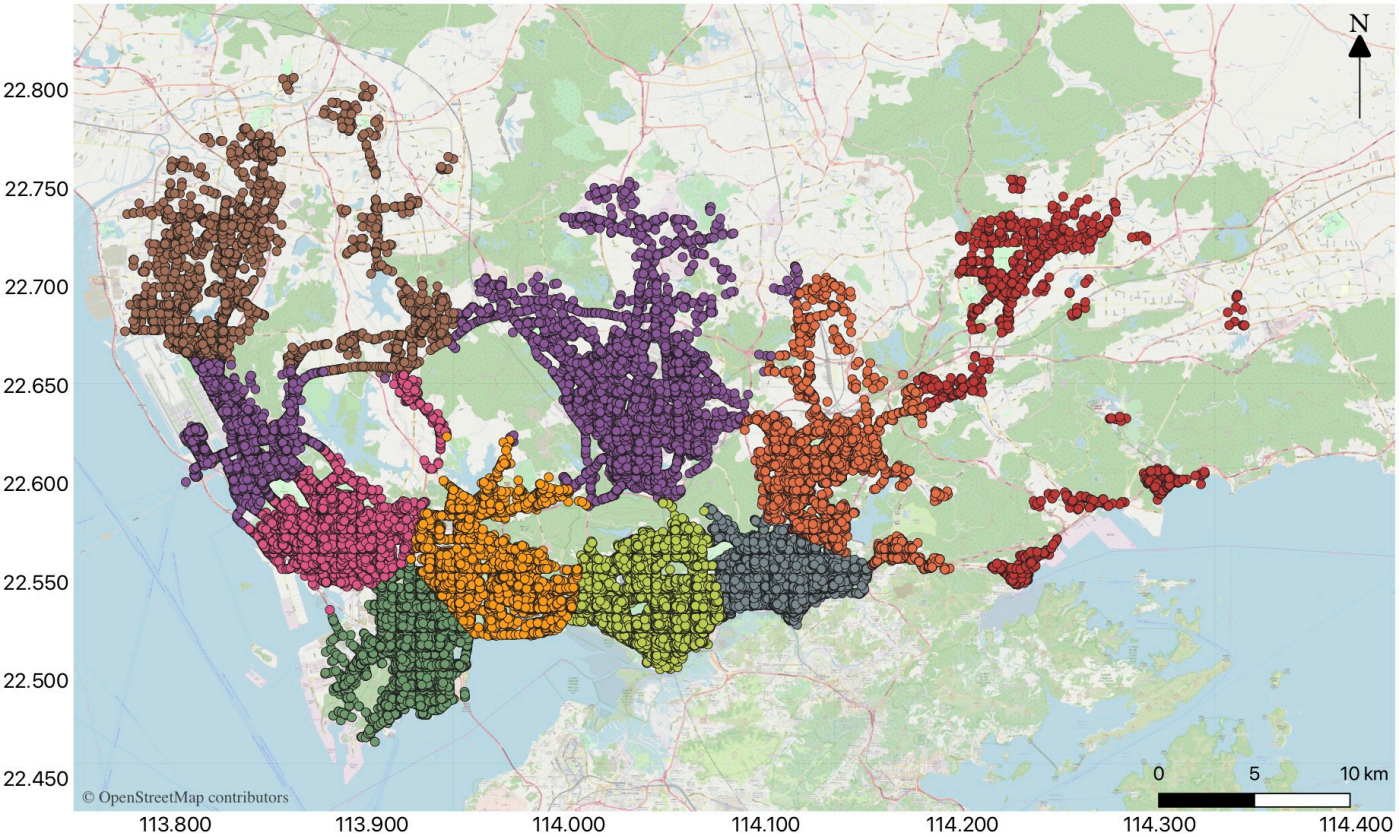
Visualization of the clustering result.

Fig 5 presents a clustering result graph, and after visualizing the clustering data, Fig 6 can be obtained.

**Fig 7:**
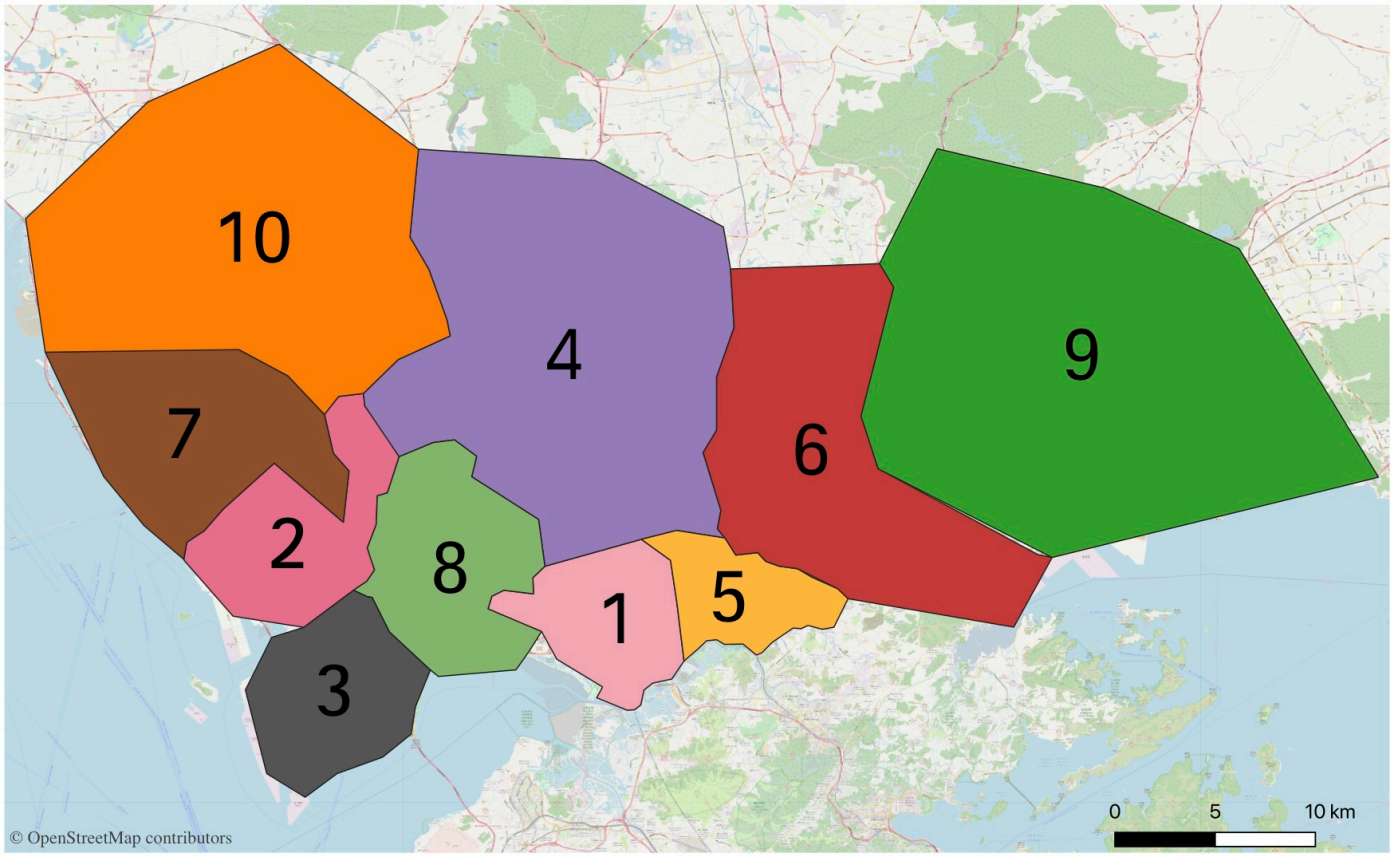
Order zones are adjusted and numbered.

The boundary of each divided zone is mainly determined by the boundary points of the each cluster. However, the boundary points were also fine-tuned based on the principle that boundaries do not cross with the main road, the rivers, the mountains or the parks. This is based on the consideration when a driver cruises near the zone boundaries to find passengers, they do not need to cross the main road, the river, the mountain or the park to pick up the passengers, which can save the cruise time. Hence, when the boundaries cross with the main road, the rivers, the mountains, or the parks, the boundaries of this part is fine-tuned to take the main road, the rivers, the mountains, or the parks as the boundaries. The order zones are fine-tuned and numbered to yield Fig 7.

### Influence variable correlation analysis

This study considers the taxi order demands to Shenzhen Airport on weekdays as an example to predict the taxi order demand to it in different zones within 60 min. We select the GPS data of all the Shenzhen taxi orders from 1 September 2015 to 31 September 2015 and calculate the order demand to the airport within a time interval of 60 min. This is followed by the correlation analysis of the factors that influence the taxi demand to the airport by a taxi.

**Fig 8:**
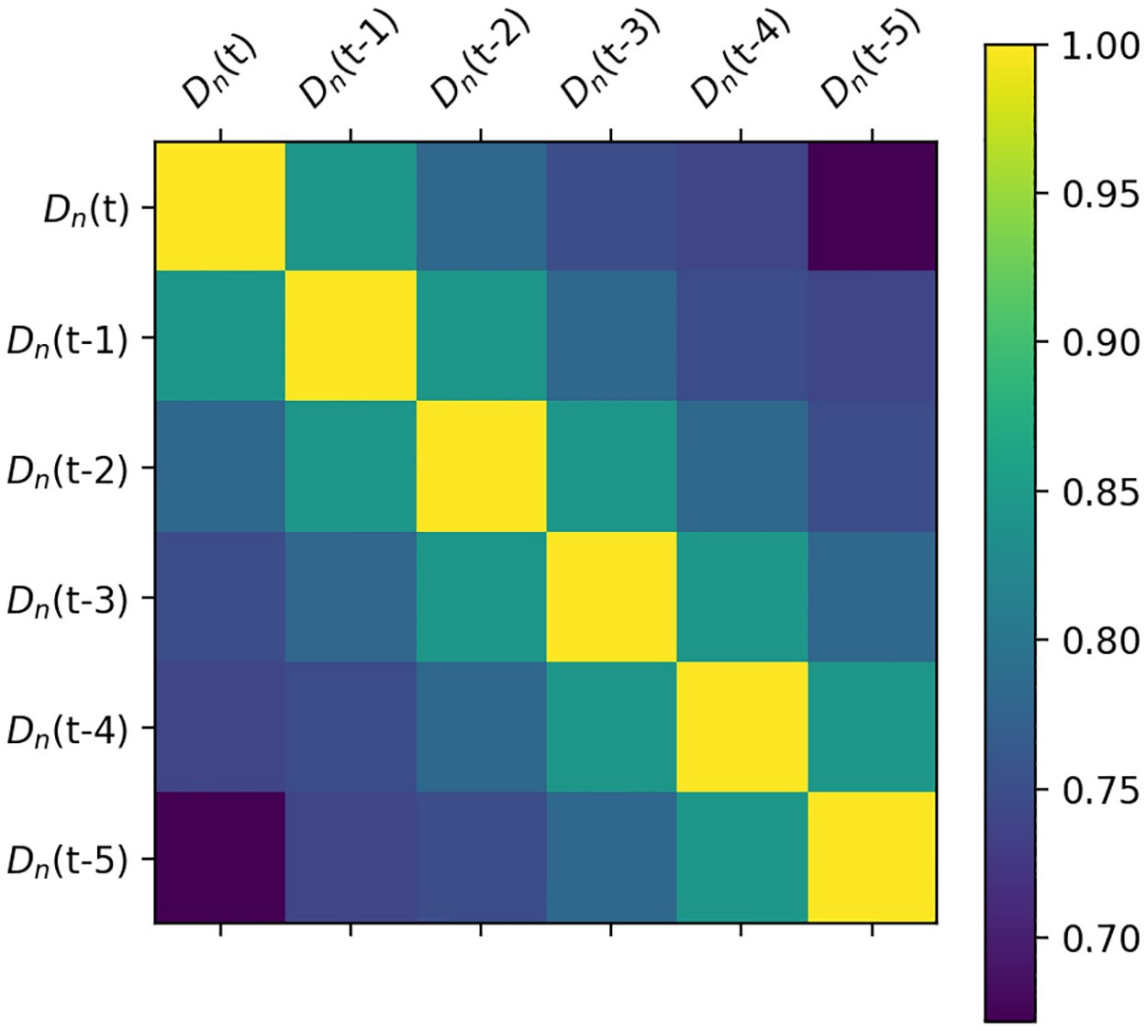
Taxi demand correlation analysis for different periods on a certain working day.

(1) The taxi demand correlation analysis of different periods on a certain working day. *D*_*n*_(*t*) is the taxi taxi demand to the airport during period [*t*, *t* + 1] of day *n*. The Pearson correlation is employed to conduct the correlation analysis. The correlation analysis is shown in Fig 8.

With 60 min as the time interval, a day can be equally divided into 24 periods. *D*_*n*_(*t* − 1) denotes the taxi order demand during period [*t* − 1, *t*), and *D*_*n*_(*t* − 2) denotes that during period [*t* − 2, *t* − 1), and so on. According to the correlation coefficients shown in Fig 8, the demand of a current period has a higher correlation with the demand of the adjacent period, and the correlations with the demand of period [*t* − 2, *t* − 1), [*t* − 1, *t*) are up to 0.75. Therefore, when predicting the taxi taxi demand, we can select *D*_*n*_(*t* − 1) and *D*_*n*_(*t* − 2) as the input variables.

**Fig 9:**
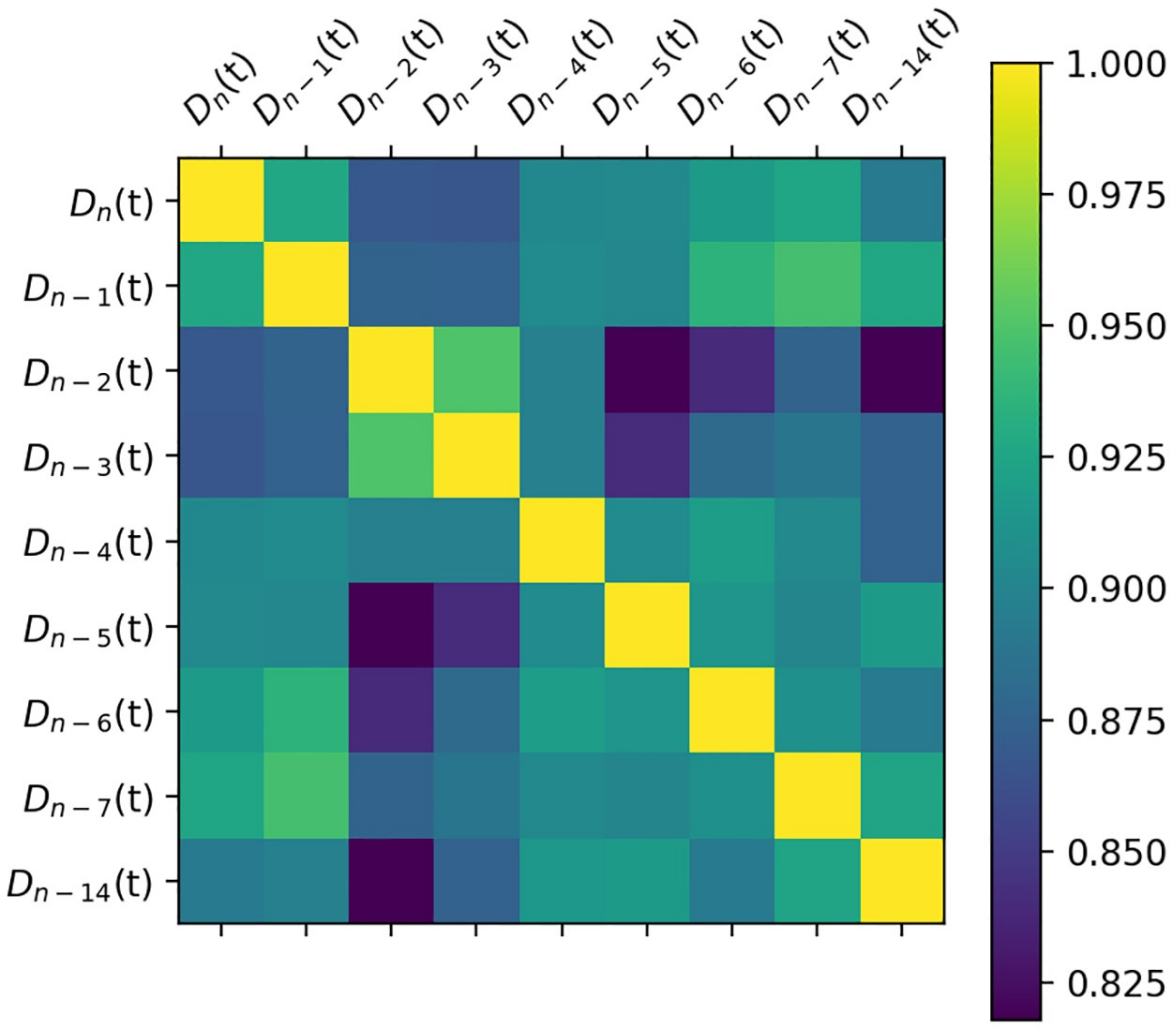
Taxi demand correlation analysis of the same period on different working days.

(2) The taxi demand correlation analysis of the same period on different days. We choose Tuesday as an example. *D*_*n*−1_(*t*) denotes the taxi demand of the previous working day, *n* −1, which is Monday, *D*_*n*−2_(*t*) denotes the taxi demand two days before, which is Sunday, and so on. The Pearson correlation is also applied to conduct the correlation analysis. The correlation analysis is shown in Fig 9.

According to the correlation coefficients in Fig 9, the correlations of the same time on different working days are quite different. The correlation between Tuesday and Saturday is around 0.86, as well as Sunday; however, the correlation with adjacent working days is as high as 0.9, and the correlation with the last Tuesday is up to 0.925. In comparison, it is slightly lower with the Tuesday before the last Tuesday. After conducting the taxi demand correlation analysis on other working days, the results show that the taxi demand correlation between working days is strong, while the correlation between working days and non-working days is weak. Therefore, when predicting the order demand, the taxi order demand of the same period on the previous 5 working days before the current period are chosen as the input variables.

**Fig 10:**
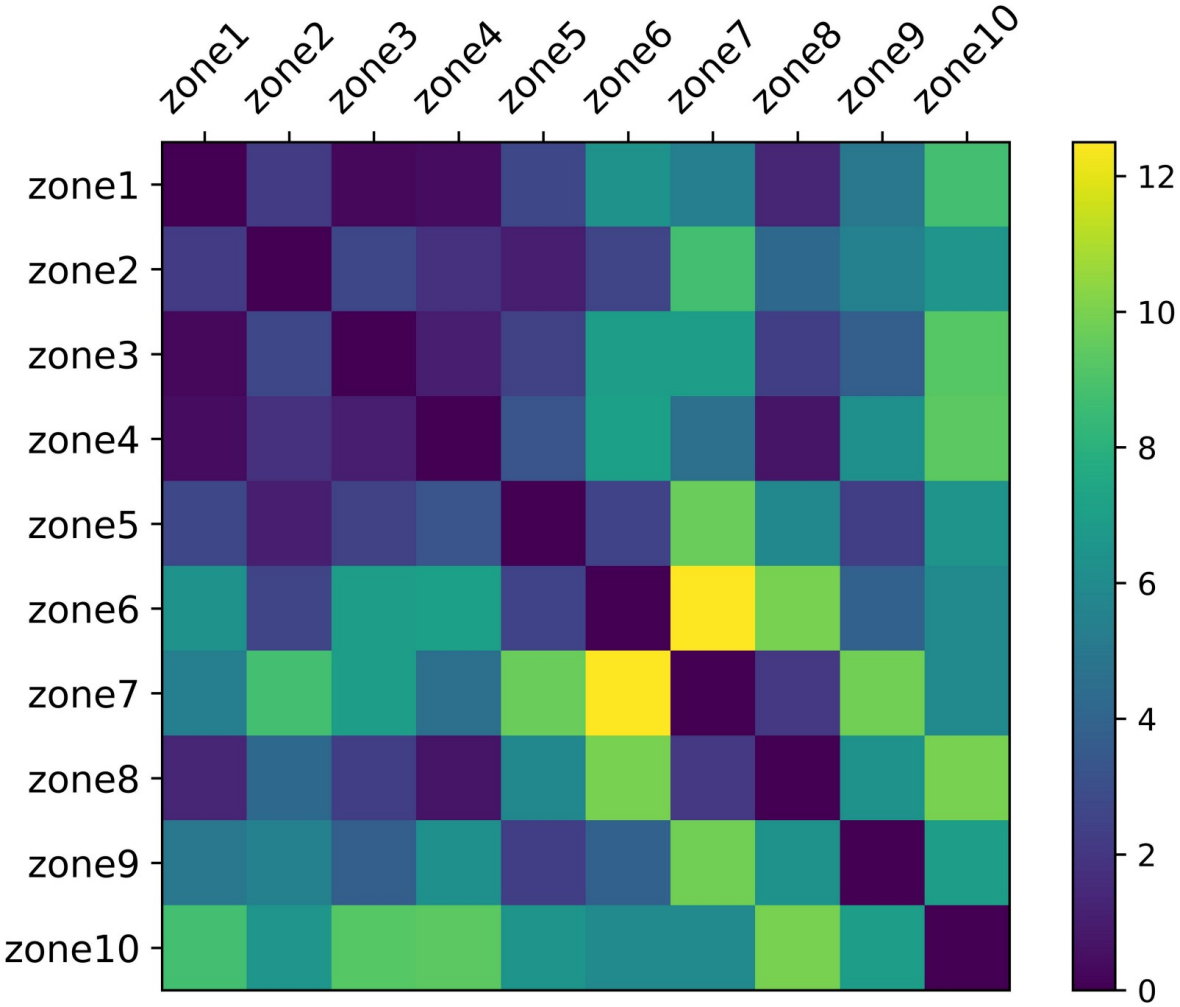
Taxi demand correlation analysis of different zones.

(3) The taxi demand correlation analysis of different zones. Intuitively, zones far away from the Shenzhen Airport generally have less taxi demand to the airport than the zones close to the airport, because the farther the distance, the more expensive the taxi fare, and thus passengers turn to other cheap transportation means, such as subway, public bus to the airport. Besides, the regularity of the taxi demand in the zones far away from the airport is also different from that of zones close to the airport. For example, passengers in zones far from the airport need to take a taxi earlier than passengers in zones close to the airport, so the peak demand for taxi orders will appear earlier in the zones far from the airports. Hence, zones far from the airport may share similar taxi demand patterns, and zones close to the airport may share similar taxi demand patterns. Meanwhile, zones with large populations and developed economies can share a similar taxi demand pattern, and zones with small populations and underdeveloped economies can share a similar taxi demand pattern. However, similar regions may not necessarily be close in space. Therefore, we employed the dynamic time warping (DTW) to measure the similarity *DTW*(*D*(*i*), *D*(*j*)) between zone *i* and zone *j*. *DTW*(*D*(*i*), *D*(*j*)) is the dynamic time warping distance between the demand patterns of two zones. *D*(*i*) denotes the taxi demand pattern of zone *i*. The smaller the *DTW*(*D*(*i*), *D*(*j*)), the more similar zone *i* and zone *j*. The details about DTW algorithm can be seen in Müller (2007). In this study, the average weekly normalized taxi demand time series of different zones spanning from September 1 to September 31 are used as the taxi demand patterns of different zones. The results are shown in Fig 10.

According to the DTW distance in Fig 10, the similarity between zones can be obtained. We define that if the value of DTW distance is less than 2, then the correlation between these two zones is strong, otherwise, the correlation is weak. If the correlation is strong, the influencing variables of the other zone are put as the input variables of the model in the zone for prediction. For example, Zone 1 is similar to Zone 3, Zone 4, and Zone 8. When predicting the taxi demand *D*_*n*_(*t*) in Zone 1, the influencing variables *D*_*n*_(*t* − 1), *D*_*n*_(*t* − 1), and the taxi order demand of the same period on the previous 5 working days before *D*_*n*_(*t*) of Zone 3, Zone 4, and Zone 8 are also added as the input variables in the prediction model. However, Zone 6, Zone 7, Zone 9, Zone 10 have weak correlation with other zones. The reason is that in these zones, there are few order demand to the airport, for most of the periods the order demand is less than 10, and there is no regularity in the demand.

### Prediction results

The data of all the working day attributes from 10 August 2015 to 18 October 2015 are selected as the training dataset, and the data of all the working day attributes from 19 October 2015 to 21 October 2015 are sorted as the testing dataset. A day is equally divided into 24 periods, and the whole area is divided into 10 zones. We define that if the order demand is less than 10 in more than 18 periods of a day in a zone, then we will not predict the order demand in this zone as people do not care about the zone where there is little demand for the whole day. Hence, the order demand in Zone 6, Zone 7, Zone 9, Zone 10 are not predicted.

**Fig 11:**
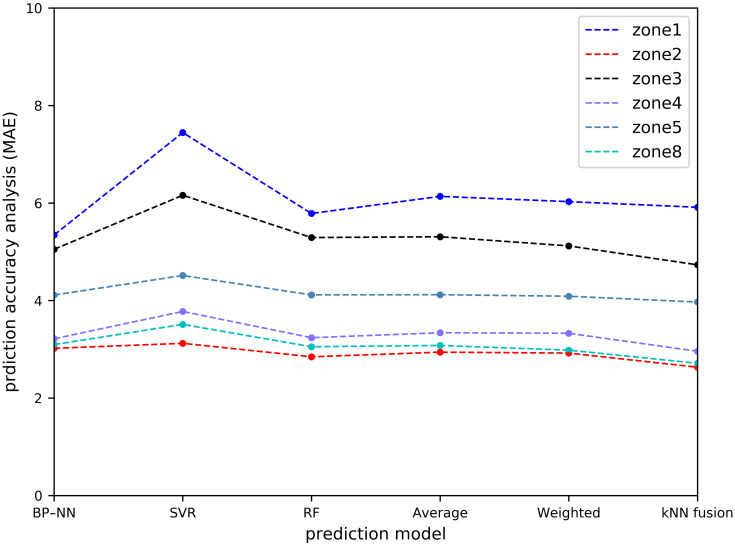
Prediction accuracy analysis with MAE.

**Fig 12:**
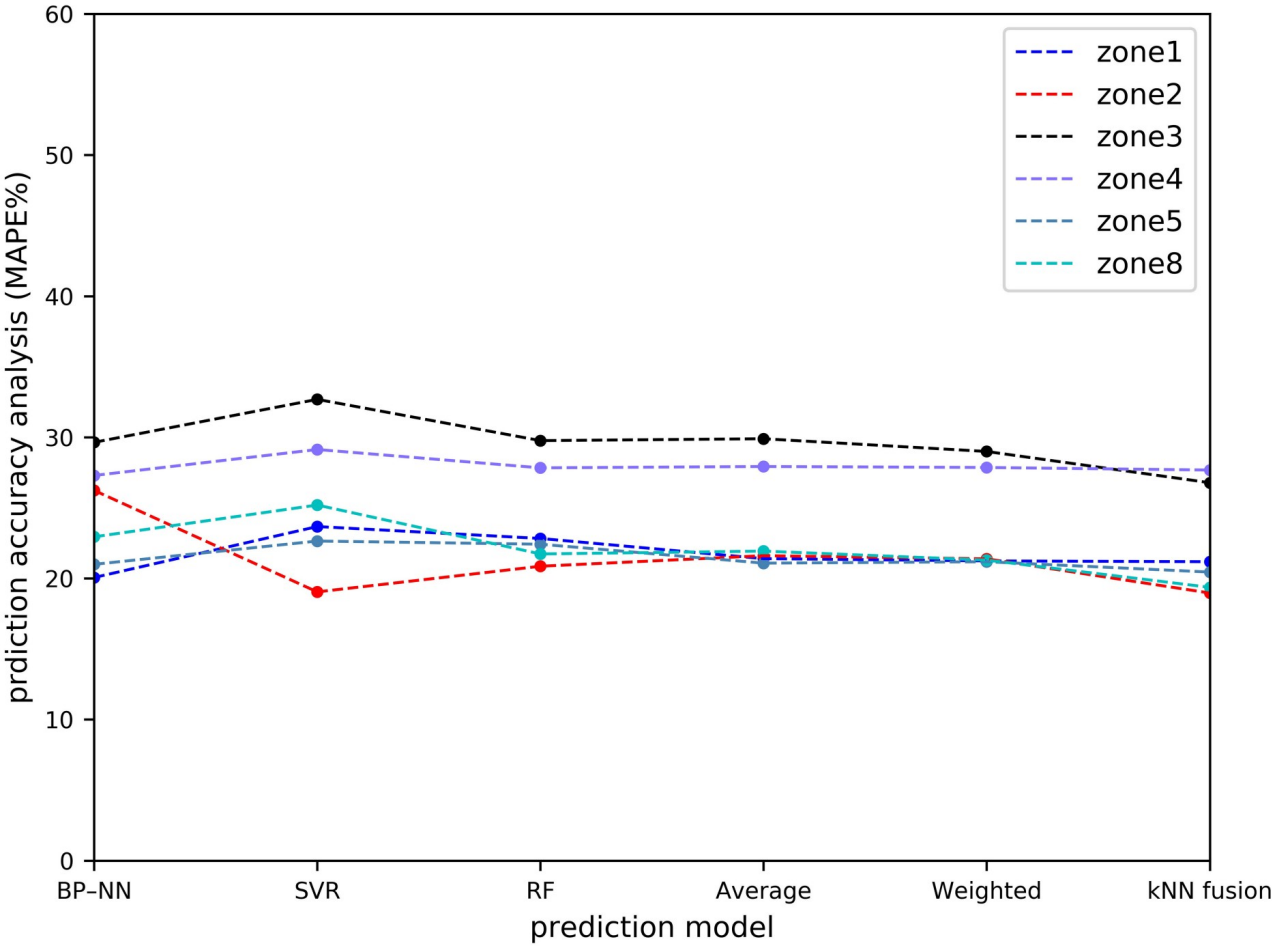
Prediction accuracy analysis with MAPE.

**Fig 13:**
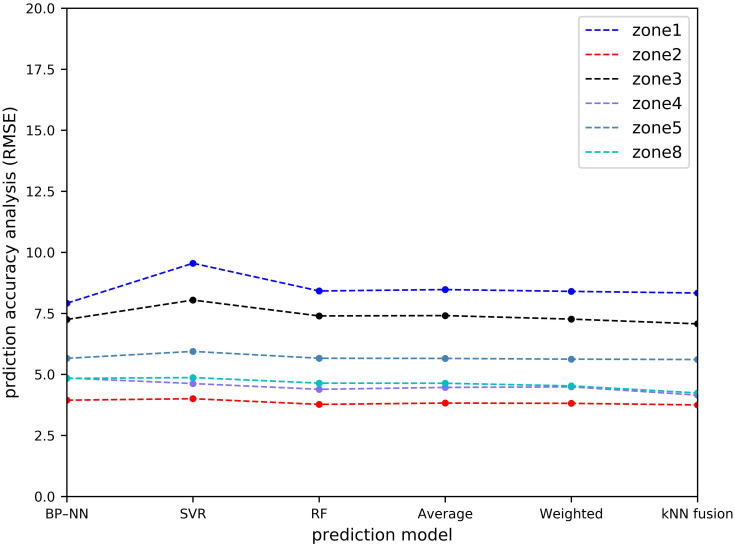
Prediction accuracy analysis with RMSE.

**Fig 14:**
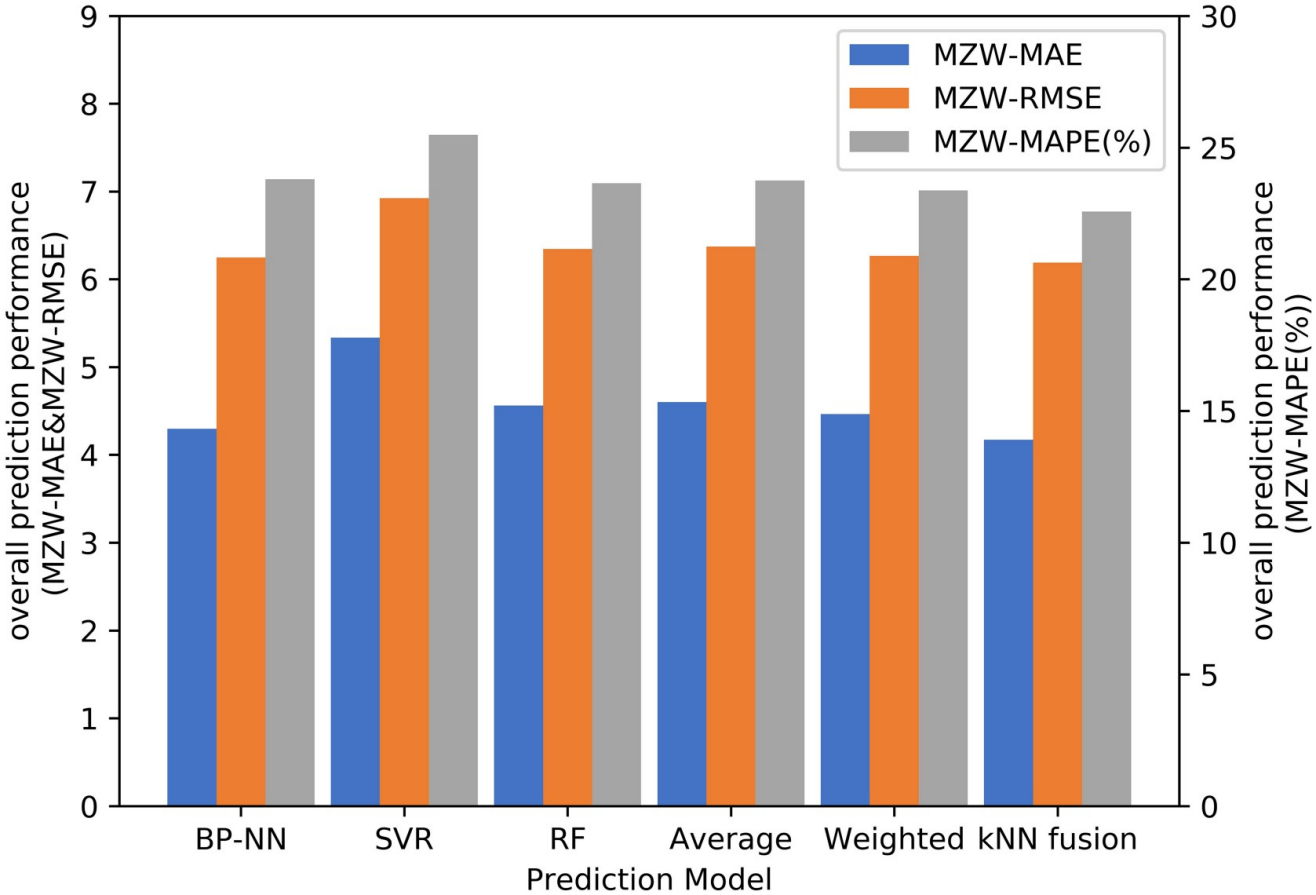
Overall prediction performance analysis.

Table 1 and Figs 11–14 show the prediction performances of these prediction models, including BP–NN, SVR, RF, average fusion-based method, weighted fusion-based method, and kNN fusion-based method in all the zones.

**Table 1:** Prediction accuracies of the different methods.

	Zone1			Zone2			Zone3		
	MAE	MAPE	RMSE	MAE	MAPE	RMSE	MAE	MAPE	RMSE
BP–NN	**5.345**	**20.063%**	**7.915**	3.019	26.236%	3.943	5.05	29.641%	7.250
SVR	7.445	23.674%	9.552	3.122	19.044%	4.004	6.159	32.686%	8.044
RF	5.785	22.831%	8.417	2.845	20.862%	3.771	5.291	29.764%	7.395
Average	6.136	21.400%	8.474	2.94	21.610%	3.826	5.308	29.895%	7.408
Weighted	6.028	21.242	8.4	2.922	21.387%	3.814	5.12	28.994%	7.264
kNN fusion	5.913	21.182%	8.334	**2.632**	**18.964%**	**3.749**	**4.732**	**26.779%**	**7.071**
	Zone4			Zone5			Zone8		
	MAE	MAPE	RMSE	MAE	MAE	RMSE	MAE	MAPE	RMSE
BP–NN	3.216	**27.293%**	4.850	4.112	20.997%	5.658	3.096	22.946%	4.836
SVR	3.775	29.133%	4.624	4.513	22.647%	5.941	3.510	25.199%	4.869
RF	3.237	27.834%	4.389	4.116	22.423%	5.660	3.052	21.733%	4.642
Average	3.339	27.929%	4.467	4.119	21.082%	5.654	3.078	21.934%	4.637
Weighted	3.328	27.859%	4.489	4.088	21.182%	5.624	2.982	21.298%	4.528
kNN fusion	**2.959**	27.679%	**4.150**	**3.969**	**20.448%**	**5.609**	**2.712**	**19.366%**	**4.240**

	MZW-MAE	MZW-MAPE	MZW-RMSE
BP–NN	4.296	23.797%	6.247
SVR	5.335	25.478%	6.923
RF	4.563	23.645%	6.346
Average	4.600	23.743%	6.374
Weighted	4.463	23.371%	6.267
kNN fusion	**4.171**	**22.569%**	**6.191**

In Zone 2, the values of the MAPE using the average and weighted fusion-based methods are 21.6% and 21.387%, whereas that using the kNN-fusion based method is 18.964%. Compared with the average and weighted fusion-based methods, the values of the MAE and RMSE using the kNN fusion-based method are relatively lower than those by other prediction models, which are 2.632 and 3.749, respectively. Similarly, the kNN fusion-based method gives the most accurate results in Zone 3 (e.g. 7.071 orders/h of the RMSE), Zone 4 (e.g. 4.150 orders/h of the RMSE), Zone 5 (e.g. 5.609 orders/h of the RMSE), and Zone 8 (e.g. 4.240 orders/h of the RMSE). In Zone 1, BP–NN is better. However, the performance of the kNN fusion-based method is also significantly better than that of the other prediction models in Zone 1. Compared with the MAPE of the average fusion-based method, the improvement of the kNN fusion-based method is 0.22%.

In terms of the overall prediction performance analysis, the values of the MZW-MAE, MZW-MAPE, and MZW-RMSE of the kNN fusion-based method are the lowest in all the six prediction methods, which are 4.171 orders/h, 22.569%, and 6.191 orders/h, respectively. This indicates that the kNN fusion-based method can give a better performance in multi-zone prediction.

### Effect of zone division

In order to investigate the performance of clustering-based zone division on order demand prediction, a comparative analysis is conducted to compare it with grid-based zone division.

**Fig 15:**
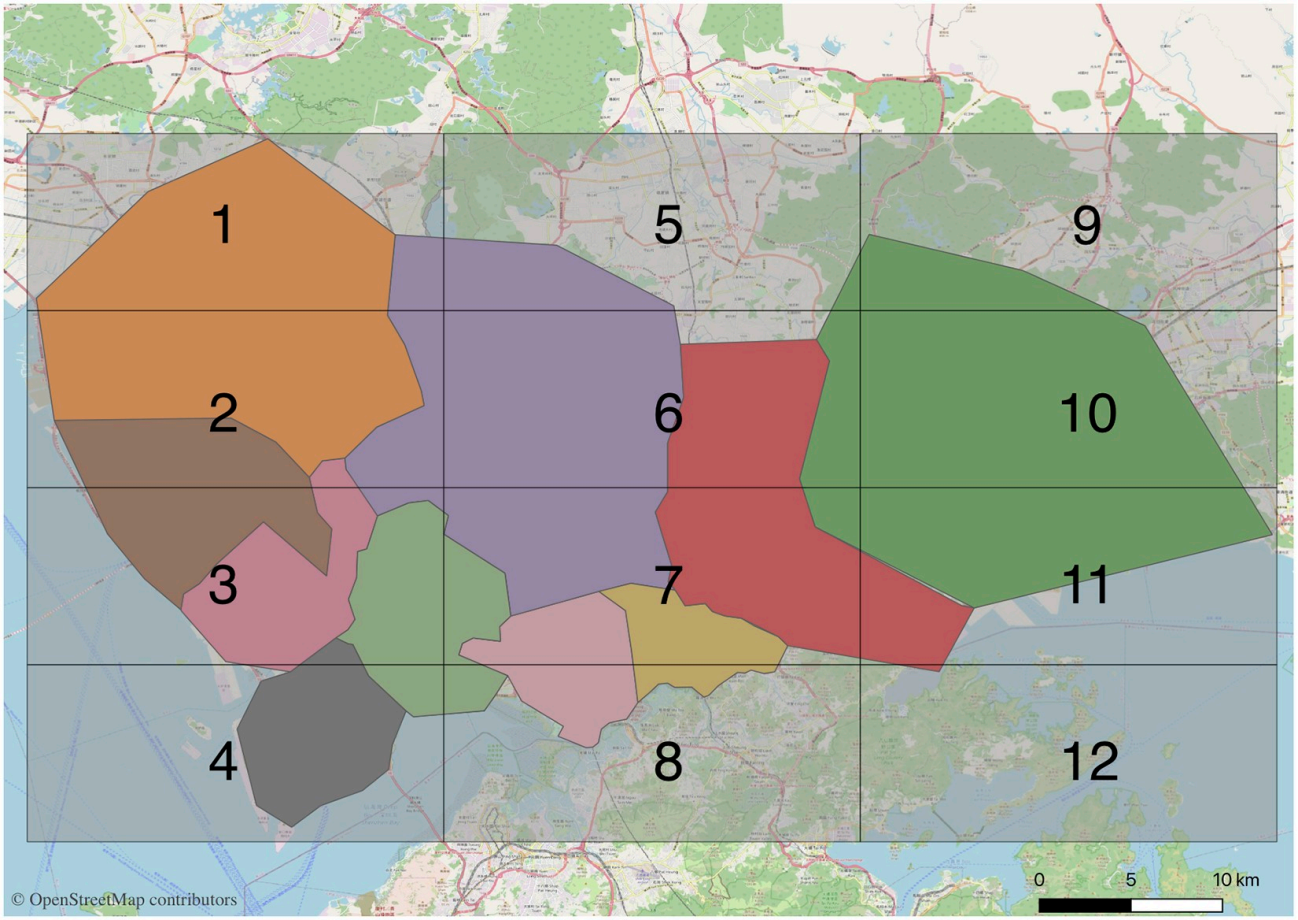
Grid zone division of the urban area in Shenzhen.

For the comparison between these two zone division methods, we should make the number of zones obtained by the two zone division methods similar. Hence, we partition the Shenzhen urban area into 3×4 grids uniformly where each grid refers to a zone (see Fig 15). Just like the taxi order demand prediction performed on clustering-based zone division, firstly, the influencing factors of different zones are analysed and the influencing variables are extracted. Subsequently, we use the same training dataset and testing dataset to train and test the models individually. The order demands in Zone 3, Zone 4, Zone 6, Zone 7, Zone 8 are less than 10 in more than 18 periods of a day, thus, these zones are not included when predicting the taxi order demand. The results are shown in Table 2.

**Table 2:** Comparison of prediction performance between the clustering-based model and the grid-based model.

	Clustering-based zone division			Grid zone division		
	MZW-MAE	MZW-MAPE	MZW-RMSE	MZW-MAE	MZW-MAPE	MZW-RMSE
BP–NN	4.296	23.797%	6.247	6.349	27.520%	9.694
SVR	5.335	25.478%	6.923	7.17	27.987%	10.086
RF	4.563	23.645%	6.346	6.362	27.158%	9.669
Average	4.600	23.743%	6.374	6.411	26.731%	9.579
Weighted	4.463	23.371%	6.267	6.271	26.698%	9.560
kNN fusion	**4.171**	**22.569%**	**6.191**	6.167	26.546%	9.505

According to the comparison results shown in Table 2, the prediction performance of the clustering-based prediction models is significantly better than those of grid-based prediction models based on the prediction accuracy indicators MZW-MAE, MZW_MAPE, and MZW_RMSE. For example, the MZW-RMSE of clustering-based kNN fusion based model is lower than the MZW-RMSE of grid-based kNN fusion based model by 2.0. This demonstrates that the proposed clustering-based model can reach a better level than the commonly used grid-based model in capturing the spatio-temporal correlation of taxi order demand.

Besides, it is also noteworthy that kNN fusion based model outperforms other prediction models under the scenario of the grid zone division. Comparing with the MAZ-MAE of the second best prediction model, the weighted fusion-based method, the improvement of the kNN fusion-based method is up to 0.1. It indicates that kNN can perform well under both the scenarios of the clustering-based zone division method and the grid zone division method.

## Conclusion

To help facilitate city-scale taxi operation management in a megacity, this paper proposes a clustering-based multi-zone order demand prediction model to divide the order zones by the K-means++ spatial clustering algorithm. The order demand is then predicted in the different divided zones based on six different prediction models: kNN fusion-based method, BP–NN, SVR, RF, average fusion-based method, and weighted fusion-based method. This study considers the taxi order demand to Shenzhen International Airport as a case study for the order zone division and the order demand prediction in different zones. The result indicates that it is effective to use the multi-zone taxi demand prediction model to divide the order zone and predict the order demands. According to the systematic comparison analysis, the kNN fusion-based method has the best overall predictive performance for multi-zone order demand based on the three multi-zone weighted indicators MZW-MAE, MZW-MAPE, and MZW-RMSE). Besides, the proposed clustering-based zone division method is compared with the commonly used grid zone division method. The results show that the prediction performances of clustering-based prediction models are significantly better than those of grid based prediction models and kNN fusion based method also outperforms other five prediction models under the scenario of grid zone division. Overall, we can support that in the case of city-scale order prediction, using the clustering-based multi-zone prediction model with the kNN-fusion based method can be effective. Moreover, it can be suggested that the multi-zone prediction model with the kNN fusion-based method proposed in this study serve as a basis of scheduling optimization at city-scale. However, limited by the data availability, our analysis has been conducted on the taxi order demand to the airport for the case study, and the divided zones are with limited number due to the demand quantity. If more sufficient data without the limitation of destination can be obtained in the future, it is suggested to consider the correlation between origins and destinations to make the zone division finer, and thus to construct a more comprehensive prediction model.
